# Distribution of Abundant and Active Planktonic Ciliates in Coastal and Slope Waters Off New England

**DOI:** 10.3389/fmicb.2017.02178

**Published:** 2017-11-14

**Authors:** Sarah J. Tucker, George B. McManus, Laura A. Katz, Jean-David Grattepanche

**Affiliations:** ^1^Department of Biological Sciences, Smith College, Northampton, MA, United States; ^2^Department of Marine Sciences, University of Connecticut, Groton, CT, United States; ^3^Program in Organismic and Evolutionary Biology, University of Massachusetts, Amherst, MA, United States

**Keywords:** RNA:DNA, depth, ciliate, oligotrichia, choreotrichia, DGGE

## Abstract

Despite their important role of linking microbial and classic marine food webs, data on biogeographical patterns of microbial eukaryotic grazers are limited, and even fewer studies have used molecular tools to assess active (i.e., those expressing genes) community members. Marine ciliate diversity is believed to be greatest at the chlorophyll maximum, where there is an abundance of autotrophic prey, and is often assumed to decline with depth. Here, we assess the abundant (DNA) and active (RNA) marine ciliate communities throughout the water column at two stations off the New England coast (Northwest Atlantic)—a coastal station 43 km from shore (40 m depth) and a slope station 135 km off shore (1,000 m). We analyze ciliate communities using a DNA fingerprinting technique, Denaturing Gradient Gel Electrophoresis (DGGE), which captures patterns of abundant community members. We compare estimates of ciliate communities from SSU-rDNA (abundant) and SSU-rRNA (active) and find complex patterns throughout the water column, including many active lineages below the photic zone. Our analyses reveal (1) a number of widely-distributed taxa that are both abundant and active; (2) considerable heterogeneity in patterns of presence/absence of taxa in offshore samples taken 50 m apart throughout the water column; and (3) three distinct ciliate assemblages based on position from shore and depth. Analysis of active (RNA) taxa uncovers biodiversity hidden to traditional DNA-based approaches (e.g., clone library, rDNA amplicon studies).

## Introduction

Planktonic marine microbes play important roles in both biogeochemical cycling pathways (Sherr and Sherr, 1984; Caron et al., 1985) and as a links between bacteria and higher trophic levels (Williams, 1981; Azam et al., 1983). Protists (microbial eukaryotes) fill complex ecological roles in part due to their trophic, morphological, genetic, and metabolic diversity (Stoecker, 1998; Worden et al., 2015; Caron, 2016). These diverse qualities shape species-environment and interspecific (predation, parasitism, etc.) interactions and thus have a significant role in the structuring of marine protist communities (Artolozaga et al., 2000; Krause, 2014; Bier et al., 2015; Fuhrman et al., 2015).

Molecular surveys have helped to reveal microbial community structure over various scales and to identify the environmental parameters driving these patterns. Much insight on marine microbial ecology and biogeography has come from DNA-based studies, a substantial majority of which have been focused on prokaryotic groups (Giovannoni et al., 1990; Delong, 1992; Long and Azam, 2001; Oakley et al., 2010; Vergin et al., 2013). For example, multi-year monitoring has revealed patterns of bacterial community composition that are predictable on the basis of seasons and environmental conditions (temperature, salinity, chlorophyll, and nutrients concentration; Treusch et al., 2009; Gilbert et al., 2011; Tinta et al., 2014; Cram et al., 2015; Sudek et al., 2015). In protists, a number of studies are beginning to reveal biotic and abiotic drivers of substantial vertical (Bachy et al., 2011; Jing et al., 2015; Massana et al., 2015; Cabello et al., 2016), horizontal (de Vargas et al., 2015; Pernice et al., 2015; Grattepanche et al., 2016b), and seasonal variation and structuring of communities (Nolte et al., 2010; Terrado et al., 2011; Kim et al., 2013). However, one limitation of DNA-based studies is that they are only informative about who is present and not the activity of these diverse lineages.

Comparisons of SSU-rRNA and SSU-rDNA have provided insight into community composition and the role of environment in shaping this community (Not et al., 2009; Logares et al., 2014; Debroas et al., 2015; Jing et al., 2015; Stecher et al., 2015; Hu et al., 2016). For example, Terrado et al. (2011) and Hu et al. (2016) showed that RNA-based libraries are more responsive to environmental conditions than DNA-based libraries. Similarly, in an experimental system, DNA-based sequence diversity did not show significant changes in response to treatments of light and prey availability, while changes in the RNA-based community correlated with experimental conditions (Charvet et al., 2014). Recent RNA-based studies of protists also suggest that low-abundance eukaryotic lineages are capable of metabolic outputs that contribute substantially to community functioning (Logares et al., 2014; Debroas et al., 2015). An advantage of using an RNA-based approach is that it allows for the distinguishing of dead or quiescent cells (e.g., those in cysts) from active cells (e.g., Stoeck et al., 2003).

Ciliates, specifically those in the Class Spirotrichea that are the focus of this study, provide a major trophic link from pico- and nanoplankton to higher trophic levels such as copepods and fish in marine systems (Sherr and Sherr, 1984; McManus and Fuhrman, 1988; Calbet and Saiz, 2005). DNA-derived studies have begun to provide a better understanding of patterns and drivers of spirotrich ciliate diversity (Countway et al., 2010; Tamura et al., 2011; Grattepanche et al., 2015). Community composition of Spirotrichea estimated by denaturing gradient gel electrophoresis (DGGE) showed that diversity closely corresponded with distance from the coast (i.e., nearshore vs. offshore), rather than environmental parameters (temperature, oxygen, depth, and chlorophyll concentration; Grattepanche et al., 2015). Using high-throughput sequencing to assess ciliate communities off the New England Coast, Grattepanche et al. (2016b) revealed that operational taxonomic unit (OTU) richness did not decrease with depth, in contrast to the declines observed below the photic zone in analyses using morphology (Wickham et al., 2011) and clone libraries (Countway et al., 2010).

Assessing the activity of ciliates in the class Spirotrichea may help to further elucidate biotic and abiotic factors that influence the distributions of ciliate communities. To examine the vertical distributions of both active and abundant ciliate communities, we assess abundant community members (SSU-rDNA) and active members (SSU-rRNA) using DGGE, a DNA community fingerprinting technique. Based on the findings of previous molecular-based studies (i.e., Debroas et al., 2015; Grattepanche et al., 2016b; Hu et al., 2016), we hypothesize that ciliate communities will be diverse and active below the photic zone and show small-scale patterns by depth throughout the water-column. Additionally, we expect that some non-abundant members of the community will have substantial contributions to metabolism (i.e., underrepresented in SSU-rDNA and highly represented in SSU-rRNA) and inversely that some abundant members of the community will not have any observed metabolic activity (highly represented in SSU-rDNA and underrepresented in SSU-rRNA).

## Methods

### Sampling and filtration

Over 2 days (August 12 and 13, 2015), we sampled at various depths at two stations off the coast of New England, USA, on board the R/V Connecticut. The first station is located in shallow waters (40 m depth; 40°59.57′ N, 71°40.96′ W) and the other one beyond the continental shelf break (1,000 m depth; 39°47.25′ N, 71°27.83′ W), hereafter referred to as nearshore and offshore stations, respectively (Figure S1). For each station, we sampled 1 L of seawater with Niskin bottles at four depths (surface, pycnocline, chlorophyll maximum depth, and deep, the latter being 35 m inshore and 400 m, the limit of the ship's hydrowire, offshore), plus additional depths between the chlorophyll maximum and 400 m at intervals of 50 m for the offshore station. Environmental parameters, including temperature, salinity, oxygen, and chlorophyll fluorescence, were measured using a CTD profiler (SeaBird Electronics, WA, USA; data accessible at http://www.bco-dmo.org/project/560529).

Serial filtrations of 1 L seawater were performed on an 80 μm nylon mesh (to remove metazoan plankton), and then on 10 and 2 μm polycarbonate filters (47 mm EMD Millipore Isopore membranes) to assess micro- and nanosize fractions, respectively. We used size fractionating (1) to remove larger organisms such as copepods and macroalgae that can suppress PCR, (2) to collect a larger number of cells by avoiding the clogging of the smaller filters, and (3) to assess the nanosize and microsize fractions, which have shown different biogeographical patterns in previous studies (Grattepanche et al., 2014, 2016a,b). The filters were cut in half using a razor blade, tweezers, and glass plate previously cleaned with RNase away. One half of the filter was immediately placed in 0.5 mL of DNA preparation buffer [100 mM NaCl, Tris-EDTA at pH 8, and 0.5% sodium dodecyl sulfate (SDS)] and stored at 4°C until DNA extraction. The other half was placed in 0.6 mL of RNeasy Lysis Buffer (Qiagen), vortexed for at least 5 min, and then flash frozen in liquid nitrogen before being moved to short-term storage in a −80°C freezer.

### Nucleic acid extraction, amplification, and sequencing

Total DNA and RNA were extracted according to the manufacturers' protocols using the Zymo Research Soil Microbe DNA MiniPrep Kit and the Qiagen RNeasy Mini Prep Kit, respectively. After RNA extraction, any potential residual DNA was further removed using Ambion TURBO DNase. Total RNA was reverse-transcribed to complementary DNA (cDNA) using the SuperScript III CellsDirect cDNA Synthesis Kit (Invitrogen) with random hexamers (Thermo Fisher Scientific). The cDNA was stored at −20°C prior to PCR.

The DNA and cDNA were amplified using PCR with SSU-rDNA primers specific to the hypervariable region 2 of the SSU rRNA gene of choreotrich and oligotrich ciliates (350 bp amplicon length; primers 152+ and 528-GC; OCSP-A from Doherty et al. (2007) and Tamura et al. (2011). PCR conditions were optimized by dilution of starting template and testing of cycling conditions in order to minimize PCR recombinants (Lahr and Katz, 2009). Twenty microliters of PCR master mix used 4 μL of Q5 Reaction buffer (NEB), 50 mM of BSA (bovine serum albumin), 50 μM of each dNTPs, 0.25 pM of each primer (152+ and 528-GC), 1 unit of Q5 Hot Star—High Fidelity DNA polymerase (NEB), and 1 μL of either DNA or cDNA template per reaction. Amplification included an initial denaturing step at 98°C for 1.5 min, 34/37 cycles of 98°C for 15 s, 59°C for 15 s, 72°C from 30 s, and a 2 min final extension at 72°C. To reduce PCR bias, five PCR products per sample were pooled prior to DGGE analyses to assess community composition. DGGEs were carried out following (Grattepanche et al., 2015). Briefly, we used 6% acrylamide gel with a denaturant gradient from 35 to 55% and ran the DGGE at 245 V for 5 min and then at 45 V for 15–16 h (see Grattepanche et al., 2015 for more details). Although some of the total and active diversity may have been lost during the process from filtration to PCR, this loss would occur randomly and thus our approach using DGGE analyses allows for robust assessments of the dominant and most active members of the community.

Despite the utility of SSU-rRNA and SSU-rDNA analyses, there are a number of limitations to this approach that need to be carefully considered (Blazewicz et al., 2013). Ribosomal rRNA accounts for the majority of the total RNA and thus provides a proxy of activity or more conservatively, the potential for protein synthesis (Blazewicz et al., 2013). Also, rRNA copy numbers vary substantially depending on metabolic state and cell size and rDNA copy number is often not related directly to the synthesis of rRNA (Raška et al., 2006; Torres-Machorro et al., 2010). Given that for our study we only assess the patterns of presence-absence of rDNA and rRNA for the most dominant and most active community members, taxa that are present in rDNA and rRNA are considered as abundant and active, those present in rDNA only as abundant, but relatively inactive, and those present in rRNA only as active, but relatively rare in the community.

### Taxonomic assessment

DGGE bands were selected based on overall coverage, band brightness, or unique position/shared position in the gel, then excised from the gels and eluted in 20 μL of 10 mM Tris buffer. For Sanger sequencing, a 1:100 dilution of eluted DNA from DGGE bands was re-amplified using the same master mix and cycling conditions except for only 30 cycles and with the non-GC clamp version of the primer set (Tamura et al., 2011). DGGE band sequence quality was evaluated by eye using SeqMan pro (DNASTAR). Unique haplotypes were identified as OTUs at a 100% sequence identity and the corresponding band labeled on DGGE gels. Using this method we assess the consistency of the sequences between SSU-rDNA and SSU-rRNA, within and between gels.

To assign taxonomy, we performed BLAST analyses through the GenBank sequence database first using only morphospecies and subsequently using environmental sequences when our sequence did not match a morphospecies (Table 1, Table S1; accessed 06/18/2016). We also constructed a gene tree of OTUs from the DGGE experiments using a curated reference alignment of morphospecies sequences from GenBank (Santoferrara et al., 2014, 2016; Grattepanche et al., 2015, 2016b). We aligned the sequences using MAFFT E (Katoh and Standley, 2013) and built the tree with the Randomized Axelerated Maximum Likelihood (RAxML) version 8 (Stamatakis, 2014) using the GTR + Gamma (rate heterogeneity) + I (invariant sites) model.

**Table 1:** Top morphospecies blast hits.

**OTU number**	**Closest morphospecies**	**Accession number**	**Percentage of similarity**	**GB number**
1	*Pelagostrobilidium neptuni*	AY541683	100	KY353249
2	*Choreotrichia* sp.	LN870020	99	KY353248
3	*Pelagostrobilidium paraepacrum*	FJ876963	96	KY353250
4	*Choreotrichia* sp.	LN870020	98	KY353251
5	*Rimostrombidium veniliae*	FJ876964	95	KY353252
6	*Rimostrombidium veniliae*	FJ876964	92	KY353253
7	*Salpingella acuminata*	EU399536	99	KY353240
8	*Salpingella acuminata*	EU399536	99	KY353239
9	*Codonellopsis nipponica*	FJ196072	98	KY353243
10	*Tintinnopsis lata*	KM982810	100	KY353242
11	*Stenosemella pacifica*	JN831794	100	KY353241
12	*Eutintinnus perminutus*	KT792926	94	KY353247
13	*Eutintinnus perminutus*	KT792926	92	KY353246
14	*Eutintinnus pectinis*	AF399170	95	KY353244
15	*Eutintinnus pectinis*	AF399170	95	KY353245
16	*Spirostrombidium subtropicum*	JN712658	96	KY353235
17	*Parastrombidinopsis minima*	DQ393786	91	KY353238
18	*Tintinnidium* sp.	JN831804	90	KY353237
19	*Parastrombidinopsis minima*	DQ393786	90	KY353236
20	*Strombidium biarmatum*	JX512970	99	KY353227
21	*Strombidium biarmatum*	JX512970	99	KY353226
22	*Strombidium biarmatum*	JX512970	98	KY353228
23	*Cyrtostrombidium* sp.	KJ534583	99	KY353229
24	*Strombidium biarmatum*	JX512970	99	KY353225
25	*Strombidium conicum*	FJ422992	99	KY353233
26	*Strombidium conicum*	FJ422992	98	KY353234
27	*Cyrtostrombidium* sp.	KJ534583	100	KY353218
28	*Strombidium paracapitatum*	KP260511	99	KY353221
29	*Strombidium paracapitatum*	KP260511	99	KY353219
30	*Strombidium biarmatum*	JX512970	100	KY353220
31	*Strombidium paracapitatum*	KP260511	98	KY353232
32	*Strombidium paracapitatum*	KP260511	99	KY353224
33	*Sinistrostrombidium cupiformum*	JX310366	97	KY353211
34	*Apostrombidium parakielum*	JX025560	96	KY353213
35	*Sinistrostrombidium cupiformum*	JX310366	97	KY353212
36	*Sinistrostrombidium cupiformum*	JX310366	98	KY353223
37	*Strombidium cf. basimorphum*	JF791016	100	KY353222
38	*Strombidium biarmatum*	JX512970	99	KY353231
39	*Cyrtostrombidium* sp.	KJ534583	99	KY353230
40	*Pseudotontonia* sp.	JX178819	99	KY353215
41	*Pseudotontonia simplicidens*	JF791015	100	KY353217
42	*Pseudotontonia* sp.	JX178819	99	KY353216
43	*Pseudotontonia* sp.	JX178819	99	KY353214

*Of 43 OTUs, 14 were 97% or less similar to known morphospecies references*.

### Statistical analyses

Community biogeography was assessed using principal coordinate analyses (PCoA), dis/similarity matrices with Jaccard or Fast Unifrac indices (Hamady et al., 2009) and a presence/absence matrix of OTUs. The Jaccard index was used to observe community patterns and Fast Unifrac for the same goal, but taking into account the phylogenetic relationship among OTUs. Analyses were performed with DNA and RNA separately and pooled together. All the statistical analyses were performed in R (version 3.3.1; R Core Team, 2016) using the Phyloseq package (version 1.16.2; McMurdie and Holmes, 2013) and Vegan (version 2.4.1, Oksanen et al., 2016) to build the rDNA tree with presence/absence, construct dissimilarity matrices, and perform PCoA.

## Results

### Environmental data

**Figure 1:**
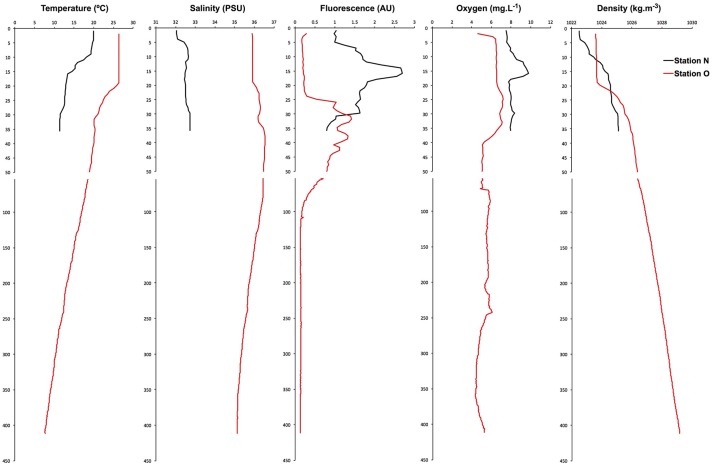
Vertical distributions of water column properties in the nearshore and offshore stations show typical summer stratification, with fresher water inshore and warmer water at shallower depths, increasing offshore. Phytoplankton biomass (as chlorophyll fluorescence) showed subsurface maxima at both stations, with the offshore station being smaller and deeper.

During the cruise, shelf waters were thermally stratified, with warm surface water overlying colder water presumably left over from deep winter mixing (Figure S2; Houghton et al., 1982). A continuous maximum in chlorophyll fluorescence extended across the shelf, deepening from about 15 m inshore to 40 m at the shelf break. Maximum phytoplankton biomass, as estimated from chlorophyll fluorescence, was about 2x higher at the inshore station (2.7 vs. 1.4 nearshore and offshore stations, respectively; arbitrary units). Salinity, dissolved oxygen, and density profiles were all typical of early summer conditions on the shelf (Figure 1). To estimate stratification at the two stations, we calculated the potential energy anomaly over the top 40 m. This quantity represents the amount of work required to mix the water column to a given depth (Simpson and Bowers, 1981; de Boer et al., 2008). The two stations showed similar levels of stratification, at 89 and 101 J/m^3^ for nearshore and offshore stations, respectively.

### Community composition: presence/absence and activity

**Figure 2:**
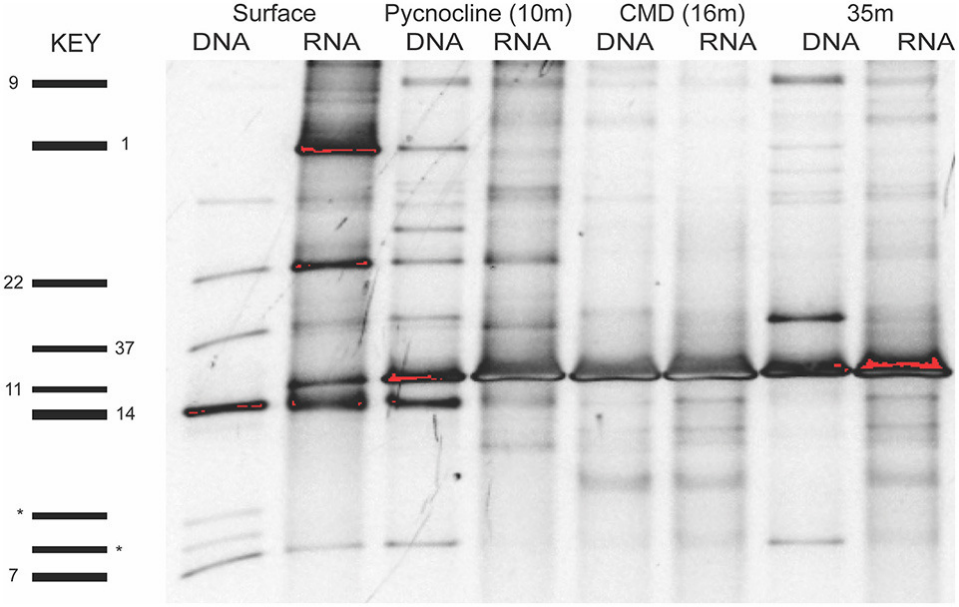
Example of a DGGE gel for the microsize fraction (>10 μm) of the nearshore station that shows that many OTUs are shared across layers and molecules (DNA and RNA). OTUs in some layers are active (RNA) but not abundant (e.g., OTU1 and OTU11, surface layer). Spirotrichea OTUs are numbered on the left side and two outgroup OTUs (dinoflagellate) are labeled by an asterisk. Analyses of OTU diversity and activity consider both the nano- and microsize fractions.

**Figure 3:**
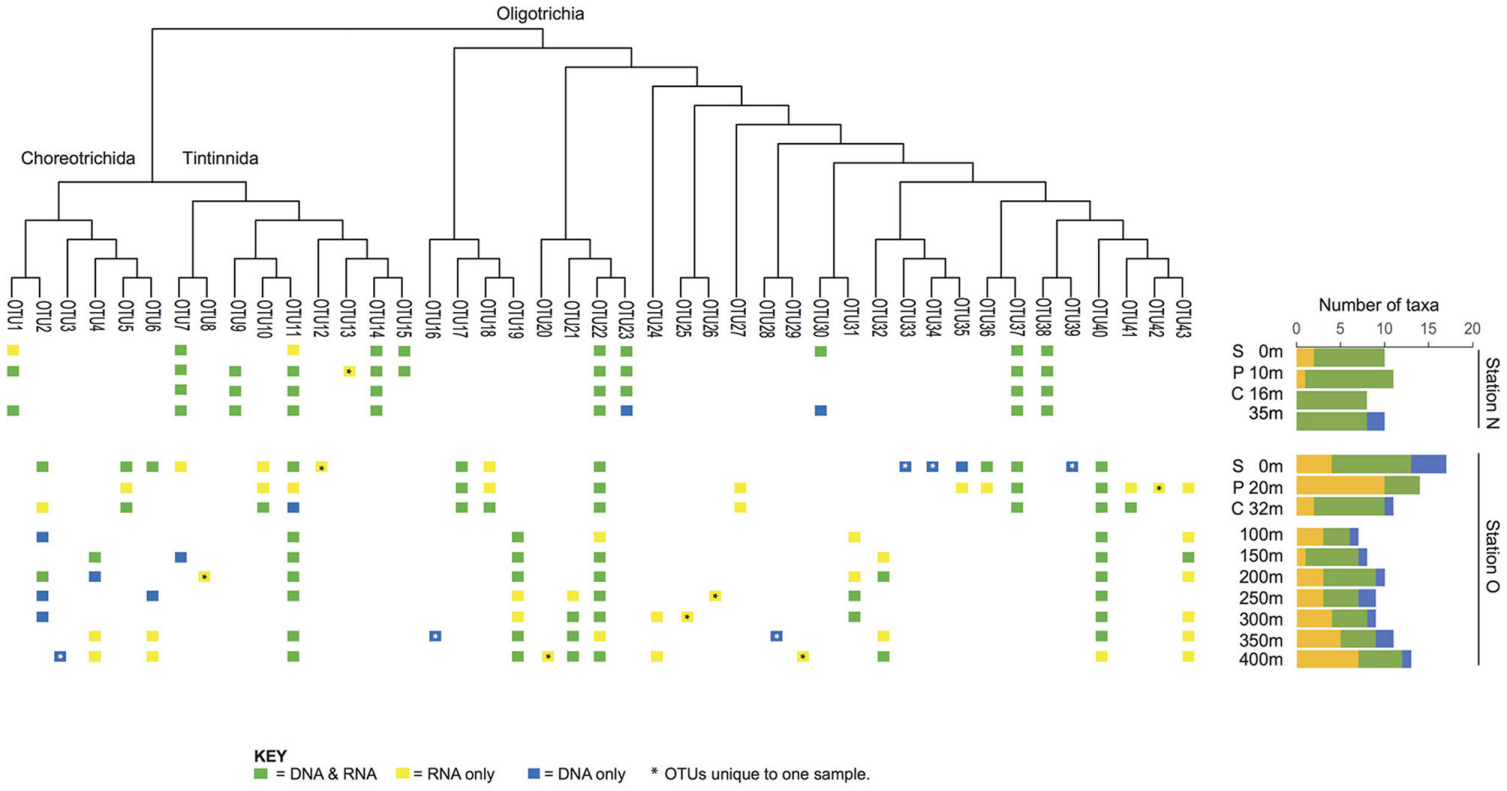
Patterns of presence/absence and activity over depth. The nearshore station (station N) shows many taxa distributed throughout the entire water column, whereas the offshore station (station O) shows more scattered distributions or distributions characterized by changes in their activity level.

The diversity and activity of spirotrich ciliates show complex patterns based on geographical position, depth, size fraction (microsize and nanosize), and molecule (DNA and RNA; Tables 2, 3, Table S2). We used DGGE to analyze a total of 56 samples: 4 and 10 depths for nearshore and offshore stations, respectively, sampled using two size fractions (2–10 μm and 10–80 μm), and considering both DNA and RNA to assess patterns of abundant (DNA) and active (RNA) community members. A total of 43 OTUs were detected from six DGGEs (Figure 2, Figures S3–S7), which include 29 OTUs represented in both molecules (DNA and RNA) in at least one sample (67% of the OTUs; Figure 3, Table 2). Of these 43 OTUs, only four were present at both stations and were generally observed in both DNA and RNA in both nano- and microsize fractions (OTUs 11, 7, 22, and 37; Figure 3, Table 2). Two of these common OTUs are distributed throughout the water column (OTUs 11 and 22) and have highest BLAST hits to *Stenosemella pacifica* (OTU11, 100%), *Salpingella acuminata* (OTU7, 99%), *Strombidium biarmatum* (OTU22, 98%), and *Strombidium cf. basimorphum* (OTU37, 100%, Table 1).

**Table 2:** OTU diversity detected in DNA, RNA, or both DNA and RNA across stations.

	**Both stations**			**Nearshore specific**			**Offshore specific**		
**Molecule**	**Both**	**DNA**	**RNA**	**BOTH**	**DNA**	**RNA**	**Both**	**DNA**	**RNA**
	4			7		1	18	6	7

*The majority of diversity is represented in station-specific OTUs*.

**Table 3:** Occurrences of OTUs detected in DNA, RNA, or both DNA and RNA across depth in the nearshore and offshore stations.

		**Both stations**			**Nearshore specific**			**Offshore specific**		
	**Molecule**	**Both**	**DNA**	**RNA**	**Both**	**DNA**	**RNA**	**Both**	**DNA**	**RNA**
	**Total**	**33**	**2**	**5**	**19**	**2**	**2**	**35**	**12**	**38**
Nearshore	Surface	3		1	5		1			
	Pycno.	4			6		1			
	CMD	4			4					
	35 m	4			4	2				
Offshore	Surface	3		1				6	4	3
	Pycno.	2		1				2		9
	CMD	2	1					6		2
	100 m	1		1				2	1	2
	150 m	2	1					4		1
	200 m	2						4	1	3
	250 m	2						2	2	3
	300 m	1						3	1	4
	350 m	1		1				3	2	4
	400 m	2						3	1	7

*The ciliates present in both stations and ciliates specific to the nearshore station tend to be both abundant and active (i.e., present in both DNA and RNA). Ciliates specific to the offshore station are commonly active, but not abundant (i.e., present in RNA and not in DNA)*.

The majority of the OTUs in the nearshore station are distributed throughout the water column (sampled to 35 m depth; Figure 3), while only three OTUs are distributed in a continuous manner throughout the water column at the offshore station (sampled to 400 m depth; Figure 3). At the nearshore station, five OTUs are consistently present in both DNA and RNA at all depths (OTUs 7, 14, 22, 38, and 37; Figure 3), and two more OTUs (OTUs 11 and 23) are present at all depths but not consistently in DNA and RNA at all depths. The offshore station has two OTUs recovered at all ten depths (OTUs 40 and 22; Figure 3). Several taxa, including OTUs 37, 17, and 5, have distributions limited to the photic zone (approximated as above the CMD), while other taxa such as OTUs 19, 21, 24, and 32 are found only below the photic zone. In addition, some OTUs are discontinuously-distributed throughout the water column. For example, OTU6 shows active and abundant cells present at the surface but then is undetected until depths of 250 m and greater (Figure 3).

A third of the OTUs (6 in DNA and 8 in RNA) are unique to a single sample (i.e., only observed in DNA or RNA at a specific size fraction and depth of a single station; Table 2) suggesting high variability of Spirotrichea in our samples. We observe unique OTUs almost exclusively in the offshore station, probably related to the higher number of samples there. These unique OTUs occur in similar numbers in DNA and RNA samples (six OTUs in DNA and eight in RNA; Figure 3, Table 2) and above and below the photic zone (six unique OTUs above the CMD and eight below; Figure 3, Table 3). These unique OTUs are more frequent in the microsize fraction than the nanosize (11 vs. 3 unique OTUs; Table S2) and from the subclass Oligotrichia (10 unique OTUs) as compared to the subclass Choreotrichia (four unique OTUs; Figure 3).

**Figure 4:**
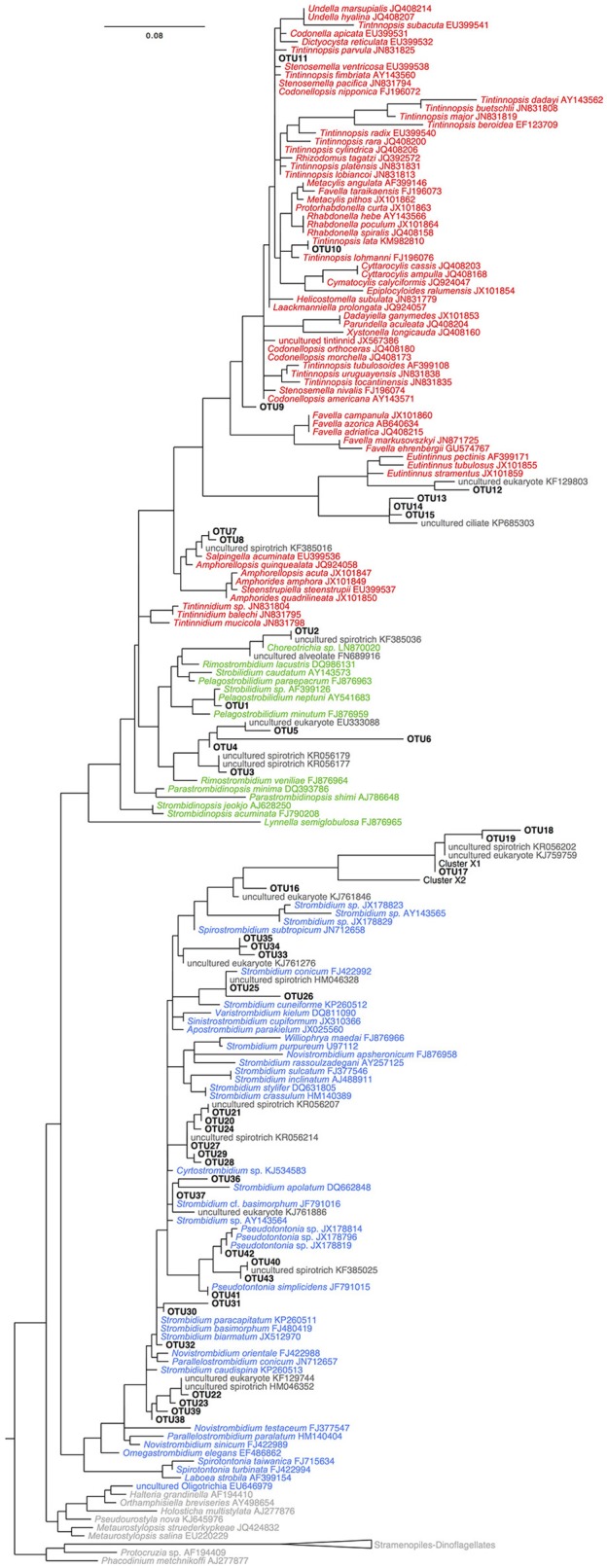
Tree of OTUs with reference sequences from GENBANK built using RAxML GTR+GAMMA+I. Colors correspond to sequences of OTUs (black), sequences of Tinntinida (red), Choreotrichida (green), and Oligotrichea (blue) morphospecies, and environmental and outgroups sequences (dark gray).

Overall 33% of the OTUs detected in this study do not have a closely related morphospecies on GenBank (i.e., sequence identity ≥97%; Table 1, Table S2). However, we find close matches for the majority of our OTUs by comparing our sequences to databases of uncultured and/or environmental sequences (Table S1). For example, OTU3 is only 96% similar to morphospecies *Pelagostrobilidium paraepacrum* FJ876963, but is 99% similar to a previously sequenced DGGE band KR056179 (Grattepanche et al., 2015). Similarly, OTU17 is only 91% similar to *Parastrombidinopsis minima* DQ393786, but 100% identical to DGGE Band KF385036 (Grattepanche et al., 2014, Table 1, Table S2). Three of these taxa, OTUs 33–35, form a distinct clade within the Oligotrichia (Figure 4), suggesting they represent undescribed lineages.

### Community biogeography

The offshore station showed higher OTU richness than the nearshore station (Figure 3). Even when comparing only the first three layers [surface (S), pycnocline (P), and chlorophyll maximum depth (CMD)], there are more OTUs in the offshore station (12 vs. 21, respectively, Figure 3). Almost all taxa are abundant and active within the nearshore station, with only 5% of nearshore taxa measured as abundant but inactive (two occurrences in DNA only over 39 total occurrences in the nearshore station; Table 3) and <10% as active but not abundant (three occurrences in RNA only over 39 total occurrences within nearshore station). In contrast, fewer taxa are both abundant and active in the offshore station (49% co-occur in DNA and RNA: 53 occurrences in both DNA and RNA over 109 total occurrences, Table 3). A few taxa in the offshore station are abundant but not active (13%, or 14 occurrences in DNA only) and an even larger portion of taxa are “rare” but active (39%, or 42 occurrences in RNA only, Figure 3, Table 3). Here, we define “rare” as undetected in DNA samples in DGGE gels and are aware that this usage is distinct from concepts of the rare biosphere that emerge from high throughput sequencing studies (Sogin et al., 2006). The “rare but active” taxa show a peak at the pycnocline and a slight increase with depth (Figure 3). Based on our reference tree (Figure 4) and BLAST results (Table 1, Table S2), oligotrich ciliates and both naked and loricate choreotrich ciliates are common in the offshore station, while oligotrich ciliates and loricate choreotrich ciliates (Tintinnida) dominated in the nearshore station, where only one naked choreotrich, related to the Strobilidiidae, was abundant (OTU1; Figure 3).

**Figure 5:**
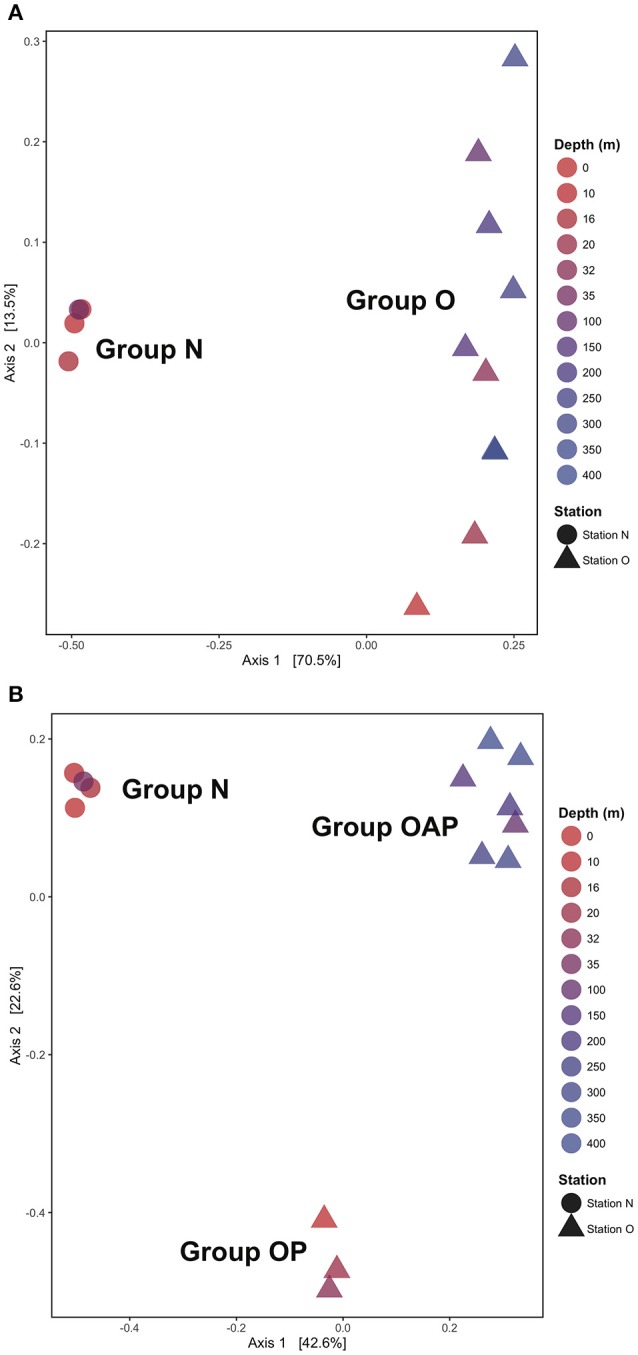
Principal Coordinates Analysis (PCoA) using **(A)** unweighted UniFrac metrics and **(B)** Jaccard index reveals two or three ciliates assemblages by stations (Group N and Group O) and by depth (Group OP and Group OAP) when phylogenic relationships among OTUs are not considered.

Principal coordinate analysis (PCoA) using a Jaccard similarity index reveals three distinct ciliate assemblages across our samples: one at the nearshore station and two at the offshore station, one above and the other below the chlorophyll maximum depth (Figure 5). Only two groups emerge when using Unifrac distances (Figure 5A), which includes information from the phylogeny of the taxa. In both approaches, samples clustered by station (Group N and Group O). Analyses performed with DNA and RNA OTUs separately (Figure S8) revealed similar patterns as analyses of the same samples with DNA and RNA OTUs pooled together (Figure 5).

## Discussion

Analyses of abundant ciliate communities at two stations 153 km apart off the coast of New England reveal: (1) a limited number of widely-distributed taxa that are both abundant and active; (2) heterogeneity of the ciliate community composition within samples taken 50 m apart through the water column at the offshore station and (3) three ciliate assemblages based on position from shore and depth. Overall, the inclusion of active (RNA) taxa reveals biodiversity hidden to traditional DNA-based approaches.

### A limited number of widely-distributed taxa are both abundant and active

Our analyses are consistent with findings of high throughput sequencing studies of marine eukaryotes (Nolte et al., 2010; Mangot et al., 2013; Logares et al., 2014; de Vargas et al., 2015). The ciliate communities sampled here consist of a few abundant, widely distributed species and a number rare lineages. For example, the OTUs common to both stations are similar to *S. biarmatum* JX512970 (OTU22), *S. cf. basimorphum* JF791016 (OTU37), *S. acuminata* EU399536 (OTU7), and *S. pacifica* JN831794 (OTU11; Tables S1, S2), all of which have been abundant in previous analyses of ciliates in the class Spirotrichea in the Northwest Atlantic (Doherty et al., 2007, 2010; Grattepanche et al., 2015). These abundant widely-distributed taxa may therefore comprise a core community of ciliates that are less impacted by environmental forces over spatial and temporal scales (Doherty et al., 2007, 2010; Dolan et al., 2009).

DGGE-based investigations of microbial communities provide insight into community dynamics and structuring and have frequently led to the detection of previously unknown microbial diversity (Pires et al., 2012; Grattepanche et al., 2015; Zhao and Xu, 2016). Our analyses using DGGE demonstrate the existence of a clade of oligotrich ciliates (OTUs 33–35) without a close match (e.g., ≥97% identity) to any previously sequenced morphospecies. This indicates that these OTUs may represent either a novel morphospecies clade or a described lineage lacking sequence data. In total, 33% of OTUs observed in this study did not have a close morphospecies sequence match on GenBank. However, the percent similarity of almost all of these OTUs improved by comparing them to uncultured or environmental sequence data (Table S1). For example, OTU17, which groups closely with OTU18 and 19, shows 100% identity to a sequenced DGGE Band (KF385033) that has been identified by deletions in its SSU-rDNA as Cluster X (Santoferrara et al., 2014; Grattepanche et al., 2016b). Cluster X has been observed repeatedly in environmental sequence libraries (Grattepanche et al., 2015, 2016b; Santoferrara et al., 2016), and was only recently identified from a preserved sample by single-cell sequencing as a *Leegardiella* sp. (Santoferrara et al., 2017). Because the majority of microbial eukaryotes are difficult to culture (Pedrós-Alió, 2006; Hoef-Emden et al., 2007) and because morphospecies are often difficult to identify from environmental samples, this disconnect between molecular and morphological analyses of ciliates is not surprising. As both environmental sequencing and cultivation/identification studies accumulate, this gap should shrink and the presence of previously unobserved clades will become clear. Our results affirm that even widely-distributed and active OTUs in a well-studied environment remain to be identified and sequenced.

### Heterogeneity throughout the water column

The heterogeneity in the composition of ciliate communities only 50 m apart in the vertical shows the dynamic nature of marine ciliates. Despite comparable mixing within the surface layers (first 40 m) at both stations (potential energy anomaly at 89 and 101 J/m^−3^, respectively), the two stations show contrasting patterns in community composition. The nearshore station presents an abundant and active community throughout its shallow water column, while the offshore station shows higher spatial heterogeneity and OTU richness. Lineages that are patchily distributed throughout the water column at the offshore station may have experienced a localized reduction in abundance or activity due to biotic (predation, viral lysis) or abiotic factors (environmental changes; Figure 3; Pedrós-Alió, 2006; Caron and Countway, 2009). The functional redundancy of taxa in the community could help to increase the stability of ecosystem functioning despite perturbations that result in changes of community composition and activity (McCann, 2000). While still little is known about the role of functional redundancy in microbial eukaryotes (Caron and Countway, 2009; Caron et al., 2012; Logares et al., 2015), rare taxa likely play a substantial role as functionally redundant community members (Lennon and Jones, 2011; Sjöstedt et al., 2012; Aanderud et al., 2015).

Recent studies also suggest that rare taxa play a significant role in environmental functioning as part of an active, rare biosphere (Pester et al., 2010; Logares et al., 2014; Debroas et al., 2015; Lawson et al., 2015). This has been reported for protists in marine environments (Logares et al., 2014) and in lakes, where the rarest OTUs represented roughly 60% of the total active biosphere (Debroas et al., 2015). A substantial portion of the active ciliate community of the offshore station in this study is represented by rare OTUs (i.e., not detected in DNA; Figure 3). Metabolically active rare lineages appear to represent a community that can respond rapidly to environmental change (Logares et al., 2014; Debroas et al., 2015; Hu et al., 2016). The high number of active but rare Spirotrichea in the offshore station, especially at the pycnocline, may thus reflect a community responding to the strong gradients associated with changes in depth (i.e., light, oxygen, temperature, biotic communities, etc.).

Our analyses revealed an increased number of rare but active ciliate taxa with increasing depth. A few previous studies also report an increased relative activity of ciliates with depth (Jing et al., 2015; Hu et al., 2016). Examining the RNA:DNA ratios of dominant taxa, Hu et al. (2016) found relative abundances of ciliate RNA sequences to increase in association with a decline in oxygen. Others studies (Stock et al., 2009; Edgcomb et al., 2011; Edgcomb and Bernhard, 2013; Jing et al., 2015) support the idea that ciliates are important grazers in oxygen minimum zones and anoxic waters. Although we observed only modest declines in oxygen with depth, the increase in non-abundant, active taxa with depth observed at the offshore station of this study may reflect the metabolic ability of some ciliate taxa to survive in deep, oxygen reduced conditions. Although our analyses did not reveal clear differences between overall patterns for the nano- and micro-size-fractionated samples, high-throughput sequencing of ciliates has found an unexpected diversity of rarer taxa from below the photic zone that is detected more commonly in the nano-size (Grattepanche et al., 2016b). Continued sampling effort using size-fractionation and analyses of rRNA and rDNA will clarify further the niches of these rare but active lineages.

### Distinct ciliate assemblages exist across our samples

Historically, studies of ciliate abundance sampled across onshore/offshore gradients have focused on tintinnids, the spirotrich clade that is both abundant in the plankton and also identifiable to species level under light microscopy. Transitions from shelf to oceanic stations have indicated a switch from species with hyaline loricae (clear) to those with agglutinated loricae (with mineral particles) and also documented changes in feeding niches as indicated by oral diameter (e.g., Beers et al., 1975; Dolan et al., 2013). Santoferrara et al. (2016) showed that patterns in morphologically-identified tintinnids generally replicated those seen with high-throughput sequencing, with distinct assemblages found inshore and offshore. In our observations, spirotrich ciliates occur in three assemblages related to distance from shore and depth—an inshore assemblage and two offshore assemblages composed of closely related OTUs separated by the chlorophyll maximum (Figure 5). The sharing of closely related OTUs in the two offshore assemblages may reflect ecologically-significant variation (i.e., eco-types) based on depth (Demir-Hilton et al., 2011; Kim et al., 2013; Logares et al., 2014; Jing et al., 2015; Cabello et al., 2016), neutral variation (e.g., interspecific, intraspecific, population variations), or both. For example, OTUs 18 and 19 are both closely related to Cluster X (Grattepanche et al., 2016b), with OTU18 detected only in the photic zone while OTU19 was only detected below the photic zone. Similarly, OTU31 and 32 are closely related to *Strombidium paracapitatum* (KP260511; 98–99% similarity) and are present in alternating patterns with depth (Figure 3), suggesting the possibility of partitioning of microhabitats (Bucci et al., 2011).

### Synthesis

Our community comparisons using both DNA and RNA capture patterns of diversity that would have been missed using DNA alone. For example, we observed that community members are both abundant and active throughout the inshore water column, while more heterogeneity existed at the offshore station, which had an increased proportion of active but rare occurrences with depth. The few OTUs that were detected across multiple depths (up to 400 m) and in both nearshore and offshore environments may form a widely-distributed, abundant, and active core ciliate community in the Northwest Atlantic that is adaptable to varying environmental factors such as changes in temperature, light, salinity, and oxygen (Dolan et al., 2009; Doherty et al., 2010). The larger number of OTUs that show site-specificity, depth-limited distributions, or changes in their activity and abundance over small depth intervals may be more susceptible to changes in biotic or abiotic conditions and thus may represent a suitable assemblage for further investigation into factors that cause community composition to be diverse (i.e., the paradox of plankton).

## Authors contributions

Conceived, designed, and performed the experiments: ST, LK, GM, and J-DG. Analyzed the data: ST, LK, GM, and J-DG. Wrote the paper: ST, LK, GM, and J-DG.

### Conflict of interest statement

The authors declare that the research was conducted in the absence of any commercial or financial relationships that could be construed as a potential conflict of interest. The reviewer PP declared a past co-authorship with one of the authors GM to the handling Editor.

## References

[B1] Aanderud Z. T. Jones S. E. Fierer N. Lennon J. T. (2015). Resuscitation of the rare biosphere contributes to pulses of ecosystem activity. Front. Microbiol. 6:24. 10.3389/fmicb.2015.00024 25688238 PMC4311709

[B2] Artolozaga I. Ayo B. Latatu A. Azúa I. Unanue M. Iriberri J. (2000). Spatial distribution of protists in the presence of macroaggregates in a marine system. FEMS Microbiol. Ecol. 33, 191–196. 10.1111/j.1574-6941.2000.tb00741.x 11098070

[B3] Azam F. F. T. Fenchel T. Field J. G. Gray J. S. Meyer L. A. Thingstad T. F. (1983). The ecological role of water-column microbes in the sea. Mar. Ecol. Prog. Ser. 257–263.

[B4] Bachy C. López-García P. Vereshchaka A. Moreira D. (2011). Diversity and vertical distribution of microbial eukaryotes in the snow, sea ice and seawater near the North Pole at the end of the polar night. Front. Microbiol. 2:106. 10.3389/fmicb.2011.00106 21833337 PMC3153057

[B5] Beers J. R. Reid F. M. H. Stewart G. L. (1975). Microplankton of the North Pacific Central Gyre. Population structure and abundance, June, 1973. Int. Rev. Hydrobiol. 60, 607–638.

[B6] Bier R. L. Bernhardt E. S. Boot C. M. Graham E. B. Hall E. K. Lennon J. T.. (2015). Linking microbial community structure and microbial processes: an empirical and conceptual overview. FEMS Microbiol. Ecol. 91:fiv113. 10.1093/femsec/fiv113 26371074

[B7] Blazewicz S. J. Barnard R. L. Daly R. A. Firestone M. K. (2013). Evaluating rRNA as an indicator of microbial activity in environmental communities: limitations and uses. ISME J. 7, 2061–2068. 10.1038/ismej.2013.102 23823491 PMC3806256

[B8] Bucci V. Nunez-Milland D. Twining B. S. Hellweger F. L. (2011). Microscale patchiness leads to large and important intraspecific internal nutrient heterogeneity in phytoplankton. Aquatic Ecol. 46, 101–118. 10.1007/s10452-011-9384-6

[B9] Cabello A. M. Latasa M. Forn I. Morán X. A. Massana R. (2016). Vertical distribution of major photosynthetic picoeukaryotic groups in stratified marine waters. Environ. Microbiol. 18, 1578–1590. 10.1111/1462-2920.13285 26971724

[B10] Calbet A. Saiz E. (2005). The ciliate-copepod link in marine ecosystems. Aquat. Microb. Ecol. 38, 157–167. 10.3354/ame038157

[B11] Caron D. A. (2016). Mixotrophy stirs up our understanding of marine food webs. Proc. Natl. Acad. Sci. 113, 2806–2808. 10.1073/pnas.1600718113 26933215 PMC4801300

[B12] Caron D. A. Countway P. D. (2009). Hypotheses on the role of the protistan rare biosphere in a changing world. Aquat. Microb. Ecol. 57, 227–238. 10.3354/ame01352

[B13] Caron D. A. Countway P. D. Jones A. C. Kim D. Y. Schnetzer A. (2012). Marine protistan diversity. Annu. Rev. Mar. Sci. 4, 467–493. 10.1146/annurev-marine-120709-142802 22457984

[B14] Caron D. A. Goldman J. C. Anderson O. K. Dennett M. R. (1985). Nutrient cycling in a microflagellate food chain: II. Population dynamics and carbon cycling. Mar. Ecol. Prog. Ser. 24, 243–254.

[B15] Charvet S. Vincent W. F. Lovejoy C. (2014). Effects of light and prey availability on Arctic freshwater protist communities examined by high-throughput DNA and RNA sequencing. FEMS Microbiol. Ecol. 88, 550–564. 10.1111/1574-6941.12324 24646212

[B16] Countway P. D. Vigil P. D. Schnetzer A. Moorthi S. D. Caron D. A. (2010). Seasonal analysis of protistan community structure and diversity at the USC Microbial Observatory (San Pedro Channel, North Pacific Ocean). Limnol. Oceanogr. 55, 2381–2396. 10.4319/lo.2010.55.6.2381

[B17] Cram J. A. Chow C. E. Sachdeva R. Needham D. M. Parada A. E. Steele J. A.. (2015). Seasonal and interannual variability of the marine bacterioplankton community throughout the water column over ten years. ISME J. 9, 563–580. 10.1038/ismej.2014.153 25203836 PMC4331575

[B18] de Boer G. J. Pietrzak J. D. Winterwerp J. C. (2008). Using the potential energy anomaly equation to investigate tidal straining and advection of stratification in a region of freshwater influence. Ocean Modell. 22, 1–11. 10.1016/j.ocemod.2007.12.003

[B19] Debroas D. Hugoni M. Domaizon I. (2015). Evidence for an active rare biosphere within freshwater protists community. Mol. Ecol. 24, 1236–1247. 10.1111/mec.13116 25690883

[B20] Delong E. F. (1992). Archaea in coastal marine environments. Proc. Natl. Acad. Sci. U.S.A. 89, 5685–5689. 1608980 10.1073/pnas.89.12.5685 PMC49357

[B21] Demir-Hilton E. Sudek S. Cuvelier M. L. Gentemann C. L. Zehr J. P. Worden A. Z. (2011). Global distribution patterns of distinct clades of the photosynthetic picoeukaryote Ostreococcus. ISME J. 5, 1095–1107. 10.1038/ismej.2010.209 21289652 PMC3146286

[B22] de Vargas C. Audic S. Henry N. Decelle J. Mahé F. Logares R.. (2015). Eukaryotic plankton diversity in the sunlit ocean. Science 348:1261605. 10.1126/science.1261605 25999516

[B23] Doherty M. Costas B. A. McManus G. B. Katz L. A. (2007). Culture-independent assessment of planktonic ciliate diversity in coastal northwest Atlantic waters. Aquat. Microb. Ecol. 48, 141–154. 10.3354/ame048141

[B24] Doherty M. Tamura M. Costas B. A. Ritchie M. E. McManus G. B. Katz L. A. (2010). Ciliate diversity and distribution across an environmental and depth gradient in Long Island Sound, USA. Environ. Microbiol. 12, 886–898. 10.1111/j.1462-2920.2009.02133.x 20113332

[B25] Dolan J. R. Landry M. R. Ritchie M. E. (2013). The species-rich assemblages of tintinnids (marine planktonic protists) are structured by mouth size. ISME J. 7, 1237–1243. 10.1038/ismej.2013.23 23426009 PMC3660678

[B26] Dolan J. R. Ritchie M. E. Tunin-Ley A. Pizay M.-D. (2009). Dynamics of core and occasional species in the marine plankton: tintinnid ciliates in the north-west Mediterranean Sea. J. Biogeogr. 36, 887–895. 10.1111/j.1365-2699.2008.02046.x

[B27] Edgcomb V. P. Bernhard J. M. (2013). Heterotrophic protists in hypersaline microbial mats and deep hypersaline basin water columns. Life 3, 346–362. 10.3390/life3020346 25369746 PMC4187137

[B28] Edgcomb V. Orsi W. Taylor G. T. Vdacny P. Taylor C. Suarez P.. (2011). Accessing marine protists from the anoxic Cariaco Basin. ISME J. 5, 1237–1241. 10.1038/ismej.2011.10 21390076 PMC3146265

[B29] Fuhrman J. A. Cram J. A. Needham D. M. (2015). Marine microbial community dynamics and their ecological interpretation. Nat. Rev. Microbiol. 13, 133–146. 10.1038/nrmicro3417 25659323

[B30] Gilbert J. A. Steele J. A. Caporaso J. G. Steinbrück L. Reeder J. Temperton B.. (2011). Defining seasonal marine microbial community dynamics. ISME J. 6, 298–308. 10.1038/ismej.2011.107 21850055 PMC3260500

[B31] Giovannoni S. J. Britschgi T. B. Moyer C. L. Field K. G. (1990). Genetic diversity in Sargasso Sea bacterioplankton. Nature 345, 60–63. 10.1038/345060a0 2330053

[B32] Grattepanche J. D. McManus G. B. Katz L. A. (2016a). Patchiness of ciliate communities sampled at varying spatial scales along the New England shelf. PLoS ONE 11:e0167659. 10.1371/journal.pone.0167659 27936137 PMC5147948

[B33] Grattepanche J. D. Santoferrara L. F. Andrade J. Oliverio A. M. McManus G. B. Katz L. A. (2014). Distribution and diversity of oligotrich and choreotrich ciliates assessed by morphology and DGGE in temperate coastal waters. Aquat. Microb. Ecol. 71, 211–221. 10.3354/ame01675

[B34] Grattepanche J. D. Santoferrara L. F. McManus G. B. Katz L. A. (2015). Distinct assemblage of planktonic ciliates dominates both photic and deep waters on the New England shelf. Mar. Ecol. Prog. Ser. 526, 1–9. 10.3354/meps11256

[B35] Grattepanche J.-D. Santoferrara L. F. McManus G. B. Katz L. A. (2016b). Unexpected biodiversity of ciliates in marine samples from below the photic zone. Mol. Ecol. 25, 3987–4000. 10.1111/mec.13745 27374257

[B36] Hamady M. Lozupone C. Knight R. (2009). Fast UniFrac: facilitating high-throughput phylogenetic analyses of microbial communities including analysis of pyrosequencing and PhyloChip data. ISME J. 4, 17–27. 10.1038/ismej.2009.97 19710709 PMC2797552

[B37] Hoef-Emden K. Küpper F. C. Andersen R. A. (2007). Meeting report: Sloan Foundation Workshop to resolve problems relating to the taxonomy of microorganisms and to culture collections arising from the barcoding initiatives; Portland ME, November 6–7, 2006. Protist 158, 135–137. 10.1016/j.protis.2007.02.001 17374505

[B38] Houghton R. W. Schlitz R. Beardsley R. C. Butman B. Chamberlin J. L. (1982). The Middle Atlantic Bight Cold Pool: evolution of the temperature structure during summer 1979. J. Phys. Oceanogr. 12, 1019–1029. 10.1175/1520-0485(1982)012<1019:TMABCP>2.0.CO;2

[B39] Hu S. K. Campbell V. Connell P. Gellene A. G. Liu Z. Terrado R.. (2016). Protistan diversity and activity inferred from RNA and DNA at a coastal ocean site in the eastern North Pacific. FEMS Microbiol. Ecol. 92:fiw050. 10.1093/femsec/fiw050 26940085

[B40] Jing H. Rocke E. Kong L. Xia X. Liu H. Landry M. R. (2015). Protist communities in a marine oxygen minimum zone off Costa Rica by 454 pyrosequencing. Biogeosci. Discuss. 12, 13483–13509. 10.5194/bgd-12-13483-2015

[B41] Katoh K. Standley D. M. (2013). MAFFT multiple sequence alignment software Version 7: improvements in performance and usability. Mol. Biol. Evol. 30, 772–780. 10.1093/molbev/mst010 23329690 PMC3603318

[B42] Kim D. Y. Countway P. D. Jones A. C. Schnetzer A. Yamashita W. Tung C.. (2013). Monthly to interannual variability of microbial eukaryote assemblages at four depths in the eastern North *Pacific*. ISME J. 8, 515–530. 10.1038/ismej.2013.173 24173457 PMC3930315

[B43] Krause S. (2014). Trait-based approaches for understanding microbial biodiversity and ecosystem functioning. Front. Microbiol. 5:251. 10.3389/fmicb.2014.00251 24904563 PMC4033906

[B44] Lahr D. J. Katz L. A. (2009). Reducing the impact of PCR-mediated recombination in molecular evolution and environmental studies using a new-generation high-fidelity DNA polymerase. BioTechniques 47, 857–866. 10.2144/000113219 19852769

[B45] Lawson C. E. Strachan B. J. Hanson N. W. Hahn A. S. Hall E. R. Rabinowitz B.. (2015). Rare taxa have potential to make metabolic contributions in enhanced biological phosphorus removal ecosystems. Environ. Microbiol. 17, 4979–4993. 10.1111/1462-2920.12875 25857222

[B46] Lennon J. T. Jones S. E. (2011). Microbial seed banks: the ecological and evolutionary implications of dormancy. Nat. Rev. Microbiol. 9, 119–130. 10.1038/nrmicro2504 21233850

[B47] Logares R. Audic S. Bass D. Bittner L. Boutte C. Christen R.. (2014). Patterns of rare and abundant marine microbial eukaryotes. Curr. Biol. 24, 813–821. 10.1016/j.cub.2014.02.050 24704080

[B48] Logares R. Mangot J.-F. Massana R. (2015). Rarity in aquatic microbes: placing protists on the map. Res. Microbiol. 166, 831–841. 10.1016/j.resmic.2015.09.009 26598213

[B49] Long R. A. Azam F. (2001). Microscale patchiness of bacterioplankton assemblage richness in seawater. Aquat. Microb. Ecol. 26, 103–113. 10.3354/ame026103

[B50] Mangot J.-F. Domaizon I. Taib N. Marouni N. Duffaud E. Bronner G.. (2013). Short-term dynamics of diversity patterns: evidence of continual reassembly within lacustrine small eukaryotes. Environ. Microbiol. 15, 1745–1758. 10.1111/1462-2920.12065 23297806

[B51] Massana R. Gobet A. Audic S. Bass D. Bittner L. Boutte C.. (2015). Marine protist diversity in European coastal waters and sediments as revealed by high-throughput sequencing. Environ. Microbiol. 17, 4035–4049. 10.1111/1462-2920.12955 26119494

[B52] McCann K. S. (2000). The diversity-stability debate. Nature 405, 228–233. 10.1038/35012234 10821283

[B53] McManus G. B. Fuhrman J. A. (1988). Control of marine bacterioplankton populations: measurement and significance of grazing. Hydrobiologia 159, 51–62. 10.1007/BF00007367

[B54] McMurdie P. J. Holmes S. (2013). phyloseq: an R Package for reproducible interactive analysis and graphics of microbiome census data. PLoS ONE 8:e61217. 10.1371/journal.pone.0061217 23630581 PMC3632530

[B55] Nolte V. Pandey R. V. Jost S. Medinger R. Ottenwälder B. Boenigk J.. (2010). Contrasting seasonal niche separation between rare and abundant taxa conceals the extent of protist diversity. Mol. Ecol. 19, 2908–2915. 10.1111/j.1365-294X.2010.04669.x 20609083 PMC2916215

[B56] Not F. del Campo J. Balagué V. de Vargas C. Massana R. (2009). New insights into the diversity of marine Picoeukaryotes. PLoS ONE 4:e7143 10.1371/journal.pone.0007143 19787059 PMC2747013

[B57] Oakley B. B. Carbonero F. van der Gast C. J. Hawkins R. J. Purdy K. J. (2010). Evolutionary divergence and biogeography of sympatric niche-differentiated bacterial populations. ISME J. 4, 488–497. 10.1038/ismej.2009.146 20054357

[B58] Oksanen J. Blanchet F. G. Friendly M. Kindt R. Legendre P. McGlinn D. (2016). Vegan: Community Ecology Package. R package version 2.4–1.

[B59] Pedrós-Alió C. (2006). Marine microbial diversity: can it be determined? Trends Microbiol. 14, 257–263. 10.1016/j.tim.2006.04.007 16679014

[B60] Pernice M. C. Giner C. R. Logares R. Perera-Bel J. Acinas S. G. Duarte C. M.. (2015). Large variability of bathypelagic microbial eukaryotic communities across the world's oceans. ISME J. 10, 945–958. 10.1038/ismej.2015.170 26451501 PMC4796934

[B61] Pester M. Bittner N. Deevong P. Wagner M. Loy A. (2010). A “rare biosphere” microorganism contributes to sulfate reduction in a peatland. ISME J. 4, 1591–1602. 10.1038/ismej.2010.75 20535221 PMC4499578

[B62] Pires A. C. Cleary D. F. Almeida A. Cunha A. Dealtry S. Mendonça-Hagler L. C. S.. (2012). Denaturing gradient gel electrophoresis and barcoded pyrosequencing reveal unprecedented archaeal diversity in mangrove sediment and rhizosphere samples. Appl. Environ. Microbiol. 78, 5520–5528. 10.1128/AEM.00386-12 22660713 PMC3406151

[B63] R Core Team (2016). R: A Language and Environment for Statistical Computing. Vienna: R Foundation for Statistical Computing Available online at: https://www.R-project.org/

[B64] Raška I. Shaw P. J. Cmarko D. (2006). New insights into nucleolar architecture and activity, in International Review of Cytology, A Survey of Cell Biology, ed Jeon K. W. (Elsevier), 255, 177–235. 10.1016/S0074-7696(06)55004-1 17178467

[B65] Santoferrara L. F. Alder V. V. McManus G. B. (2017). Phylogeny, classification and diversity of Choreotrichia and Oligotrichia (Ciliophora, Spirotrichea). Mol. Phylogenet. Evol. 112, 12–22. 10.1016/j.ympev.2017.03.010 28286224

[B66] Santoferrara L. F. Grattepanche J.-D. Katz L. A. McManus G. B. (2014). Pyrosequencing for assessing diversity of eukaryotic microbes: analysis of data on marine planktonic ciliates and comparison with traditional methods. Environ. Microbiol. 16, 2752–2763. 10.1111/1462-2920.12380 24444191

[B67] Santoferrara L. F. Grattepanche J.-D. Katz L. A. McManus G. B. (2016). Patterns and processes in microbial biogeography: do molecules and morphologies give the same answers? ISME J. 10, 1779–1790. 10.1038/ismej.2015.224 26849313 PMC4918432

[B68] Sherr B. F. Sherr E. B. (1984). Role of heterotrophic protozoa in carbon and energy flow in aquatic ecosystems, in Current Perspectives in Microbial Ecology, eds Klug M. J. Reddy C. A. (Washington, DC: Marine Microbial Food Webs), 412–423.

[B69] Simpson J. H. Bowers D. (1981). Models of stratification and frontal movement in shelf seas. Deep Sea Res. A Oceanogr. Res. Pap. 28, 727–738. 10.1016/0198-0149(81)90132-1

[B70] Sjöstedt J. Koch-Schmidt P. Pontarp M. Canbäck B. Tunlid A. Lundberg P.. (2012). Recruitment of members from the rare biosphere of marine bacterioplankton communities after an environmental disturbance. Appl. Environ. Microbiol. 78, 1361–1369. 10.1128/AEM.05542-11 22194288 PMC3294490

[B71] Sogin M. L. Morrison H. G. Huber J. A. Mark Welch D. Huse S. M. Neal P. R.. (2006). Microbial diversity in the deep sea and the underexplored “rare biosphere.” Proc. Natl. Acad. Sci. U.S.A. 103, 12115–12120. 10.1073/pnas.0605127103 16880384 PMC1524930

[B72] Stamatakis A. (2014). RAxML version 8: a tool for phylogenetic analysis and post-analysis of large phylogenies. Bioinformatics 30, 1312–1313. 10.1093/bioinformatics/btu033 24451623 PMC3998144

[B73] Stecher A. Neuhaus S. Lange B. Frickenhaus S. Beszteri B. Kroth P. G. (2015). rRNA and ribosomal RNA gene based assessment of sea ice protist biodiversity from the central Arctic Ocean. Eur. J. Phycol. 51, 31–46. 10.1080/09670262.2015.1077395

[B74] Stock A. Jürgens K. Bunge J. Stoeck T. (2009). Protistan diversity in suboxic and anoxic waters of the Gotland Deep (Baltic Sea) as revealed by 18S rRNA clone libraries. Aquat. Microb. Ecol. 55, 267–284. 10.3354/ame01301

[B75] Stoeck T. Fowle W. H. Epstein S. S. (2003). Methodology of protistan discovery: from rRNA detection to quality scanning electron microscope images. Appl. Environ. Microbiol. 69, 6856–6863. 10.1128/AEM.69.11.6856-6863.2003 14602650 PMC262282

[B76] Stoecker D. K. (1998). Conceptual models of mixotrophy in planktonic protists and some ecological and evolutionary implications. Eur. J. Protistol. 34, 281–290. 10.1016/S0932-4739(98)80055-2

[B77] Sudek S. Everroad R. C. Gehman A. L. Smith J. M. Poirier C. L. Chavez F. P.. (2015). Cyanobacterial distributions along a physico-chemical gradient in the Northeastern Pacific Ocean. Environ. Microbiol. 17, 3692–3707. 10.1111/1462-2920.12742 25522910

[B78] Tamura M. Katz L. A. McManus G. B. (2011). Distribution and diversity of oligotrich and choreotrich ciliates across an environmental gradient in a large temperate estuary. Aquat. Microb. Ecol. 64, 51–67. 10.3354/ame01509

[B79] Terrado R. Medrinal E. Dasilva C. Thaler M. Vincent W. F. Lovejoy C. (2011). Protist community composition during spring in an Arctic flaw lead polynya. Polar Biol. 34, 1901–1914. 10.1007/s00300-011-1039-5

[B80] Tinta T. Vojvoda J. Mozetič P. Talaber I. Vodopivec M. Malfatti F.. (2014). Bacterial community shift is induced by dynamic environmental parameters in a changing coastal ecosystem (northern Adriatic, northeastern Mediterranean Sea) - a 2-year time-series study. Environ. Microbiol. 17, 3581–3596. 10.1111/1462-2920.12519 24903068

[B81] Torres-Machorro A. L. Hernández R. Cevallos A. M. López-Villaseñor I. (2010). Ribosomal RNA genes in eukaryotic microorganisms: witnesses of phylogeny? FEMS Microbiol. Rev. 34, 59–86. 10.1111/j.1574-6976.2009.00196.x 19930463

[B82] Treusch A. H. Vergin K. L. Finlay L. A. Donatz M. G. Burton R. M. Carlson C. A.. (2009). Seasonality and vertical structure of microbial communities in an ocean gyre. ISME J. 3, 1148–1163. 10.1038/ismej.2009.60 19494846

[B83] Vergin K. L. Done B. Carlson C. A. Giovannoni S. J. (2013). Spatiotemporal distributions of rare bacterioplankton populations indicate adaptive strategies in the oligotrophic ocean. Aquat. Microb. Ecol. 71, 1–13. 10.3354/ame01661

[B84] Wickham S. A. Steinmair U. Kamennaya N. (2011). Ciliate distributions and forcing factors in the Amundsen and Bellingshausen Seas (Antarctic). Aquat. Microb. Ecol. 62, 215–230. 10.3354/ame01468

[B85] Williams I. P. J. (1981). Incorporation of Microheterotrophic Processes into the Classical Paradigm of the Planktonic Food Web. Kiel: Kieler Meeresforsch.

[B86] Worden A. Z. Follows M. J. Giovannoni S. J. Wilken S. Zimmerman A. E. Keeling P. J. (2015). Environmental science. Rethinking the marine carbon cycle: factoring in the multifarious lifestyles of microbes. Science 347:1257594. 10.1126/science.1257594 25678667

[B87] Zhao F. Xu K. (2016). Biodiversity patterns of soil ciliates along salinity gradients. Eur. J. Protistol. 53, 1–10. 10.1016/j.ejop.2015.12.006 26773903

