# Supplementary material for: Distribution of Abundant and Active Planktonic Ciliates in Coastal and Slope Waters Off New England
Source: Front Microbiol. 2017 Nov 14;8:2178. doi: 10.3389/fmicb.2017.02178 (PMC5715329; doi:10.3389/fmicb.2017.02178)
Supplement: Supplementary file 1 [file DataSheet1.PDF]

Supplementary Material

Distribution of abundant and active planktonic ciliates in coastal and slope waters off New England

Sarah J. Tucker, George B. McManus, Laura A. Katz, Jean-David Grattepanche*

*** Correspondence:** Jean-David Grattepanche: jgrattepanche@smith.edu

Table S1. All OTUs with higher similarity to an uncultured sequence than to a morphospecies reference (Table 1). Almost all OTUs without a close morphospecies match (97% or less similar) had a higher similarity to an uncultured sequence (except OTU33 and 35), and are here denoted by an *.

OTU #	Closest BLAST	Accession #	Similarity	GB #
2	DGGE Band	KF385036	100	KY353248
3*	DGGE Band	KR056179	99	KY353250
5*	Uncultured eukaryote clone	EU333088	99	KY353252
6*	Uncultured alveolate clone	FN689916	93	KY353253
7	DGGE Band	KF385016	100	KY353240
12*	Uncultured eukaryote clone	KF129803	97	KY353247
13*	Uncultured ciliate clone	KP685303	97	KY353246
14*	Uncultured ciliate clone	KP685303	100	KY353244
15*	Uncultured ciliate clone	KP685303	98	KY353245
16*	Uncultured eukaryote clone	KJ761846	97	KY353235
17*	DGGE Band	KF385036	100	KY353238
18*	Uncultured eukaryote clone	KJ759759	97	KY353237
19*	DGGE Band	KR056202	98	KY353236
21	DGGE Band	KR056207	100	KY353226
22	Uncultured eukaryote clone	KF129744	100	KY353228
25	DGGE Band	HM046328	100	KY353233
26	DGGE Band	HM046328	99	KY353234
27	DGGE Band	KR056214	100	KY353218
34*	Uncultured eukaryote clone	KJ761276	97	KY353213
40	DGGE Band	KF385025	100	KY353215

Table S2. Analyses of occurrences across samples reveals that ciliates present in both nearshore and offshore stations tend to be intermediate size and both abundant and active (i.e. present in both DNA and RNA); station-specific ciliates have more complex patterns.

		Total Occurrences 148									Nearshore Total 39									Offshore Total 109								
		Both stations 40									Nearshore Specific 23									Offshore Specific 85								
	Molecule	Both 33			DNA 2			RNA 5			Both 19			DNA 2			RNA 2			Both 35			DNA 12			RNA 38		
	Size	n	b	m	n	b	m	n	b	m	n	b	m	n	b	m	n	b	m	n	b	m	n	b	m	n	b	m
Nearshore	Surface		3						1		4	1							1									
	Pycno.		4								3	1	2						1									
	CMD		4								2	1	1															
	35m		4								1	1	2	2														
Offshore	Surface		3						1											2	2	2			4	2		1
	Pycno.		2						1											1	1					5		4
	CMD		2			1														5	1					2		
	100m		1						1											1	1		1			1		1
	150m		2			1														2	1	1				1		
	200m		2																	3	1		1			2		1
	250m		2																	1	1		1	1		2		1
	300m		1																	2	1		1			1		3
	350m		1						1											2	1		1		1	2	1	1
	400m		2																	3					1	2	2	3
	Total		33			2			5		10	4	5	2					2	22	10	3	5	1	6	20	3	15

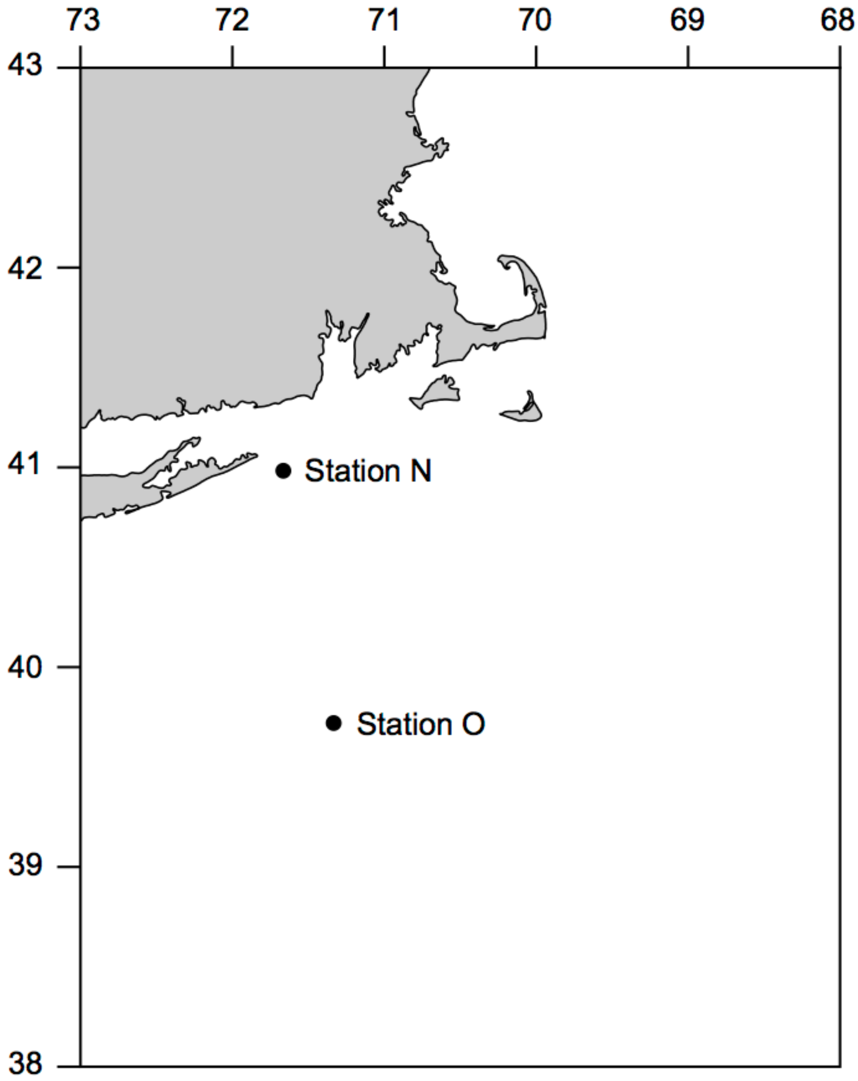

Figure S1. Map of nearshore (43km from shore) and offshore stations (135km off shore).

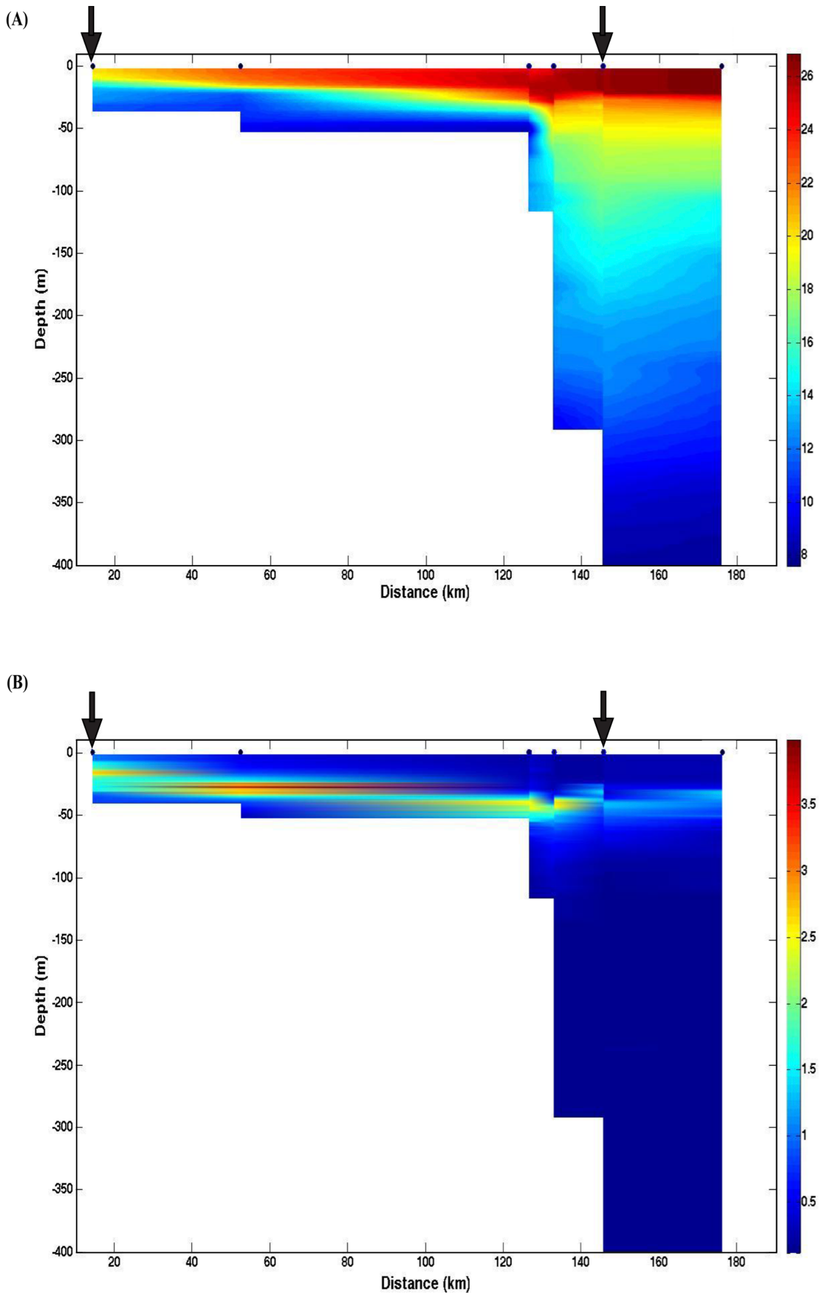

Figure S2. Environmental structuration. **(A)** Environmental structuration by temperature (°C) and **(B)** environmental structuration by chlorophyll concentration (fluorescence in arbitrary units) during the 2015 cruise from Avery Point, CT, USA. Samples were taken at a nearshore station (43km from shore) and an offshore, slope station (135km off shore) as shown with arrows.

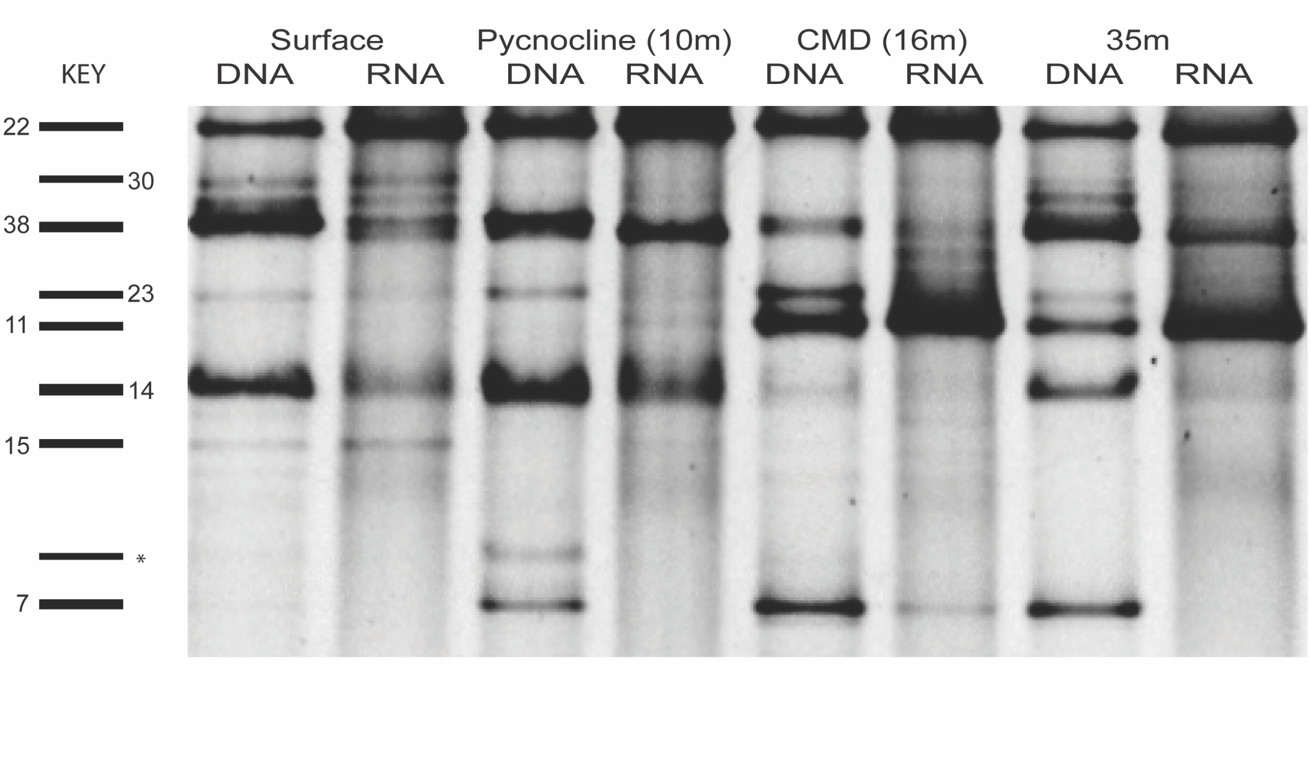

Figure S3. Nearshore station nanosize (2-10μm) DNA and RNA samples.

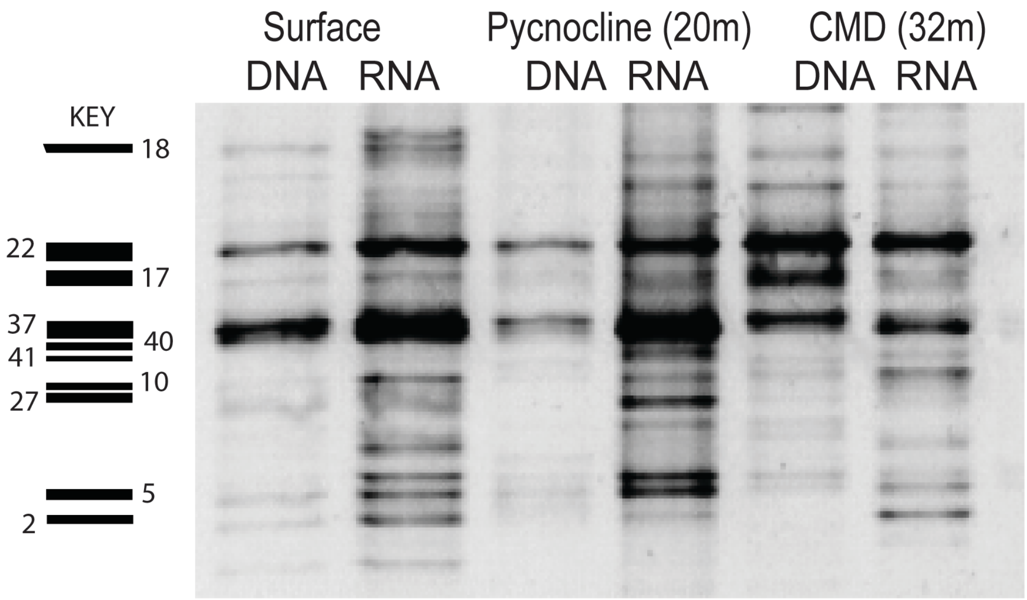

Figure S4. Offshore nanosize fraction (2-10µm) DNA and RNA samples of photic layers.

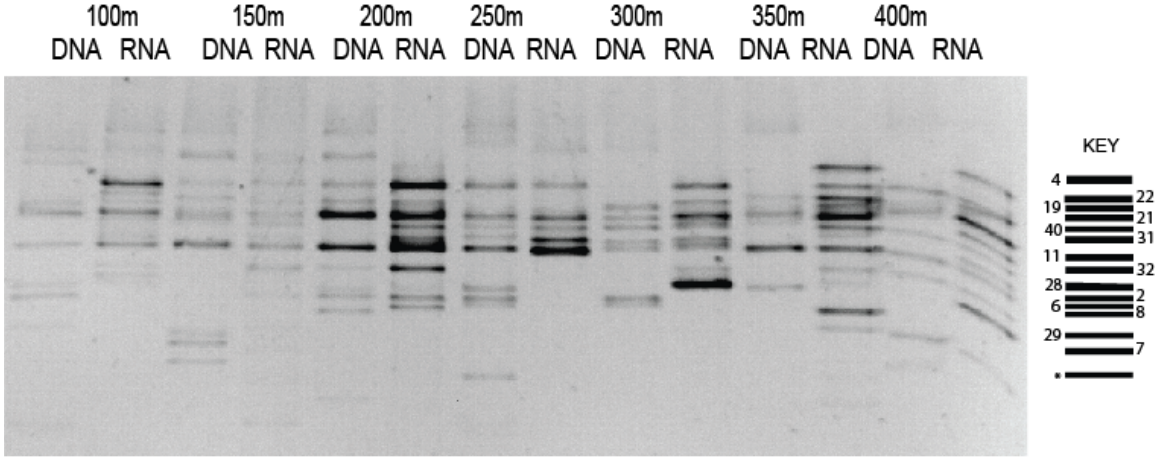

Figure S5. Offshore station nanosize fraction (2-10µm) DNA and RNA samples of aphotic layers. * represents an outgroup of dinoflagellate.

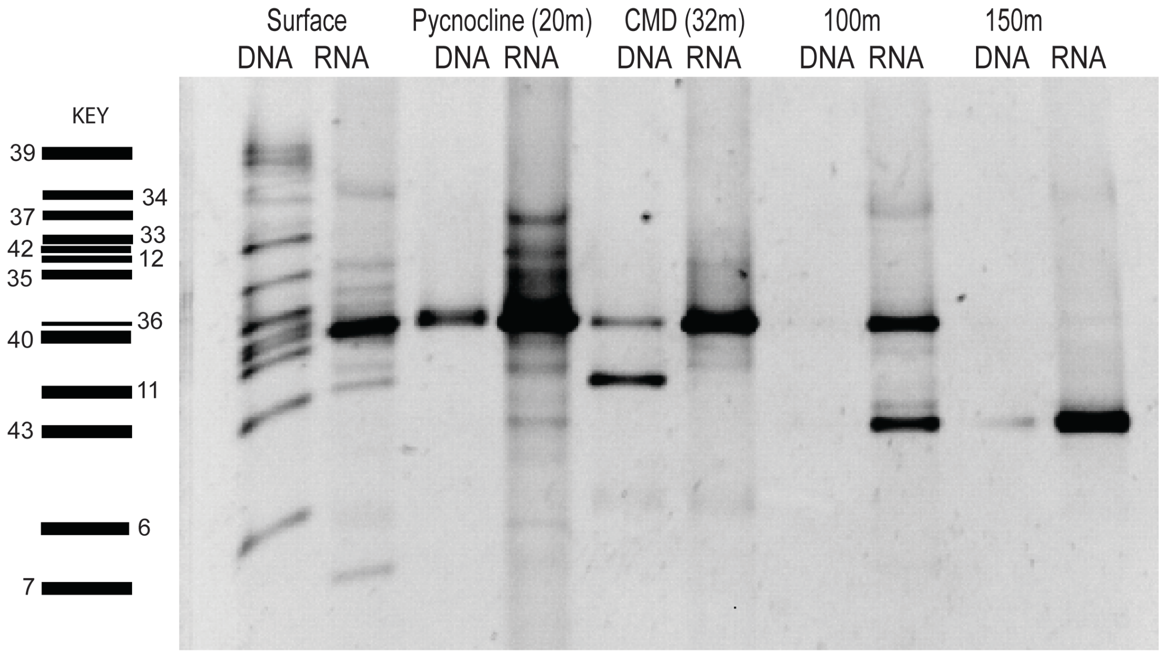

Figure S6. Offshore microsize (>10μm) DGGE of shallow layers DNA and RNA samples.

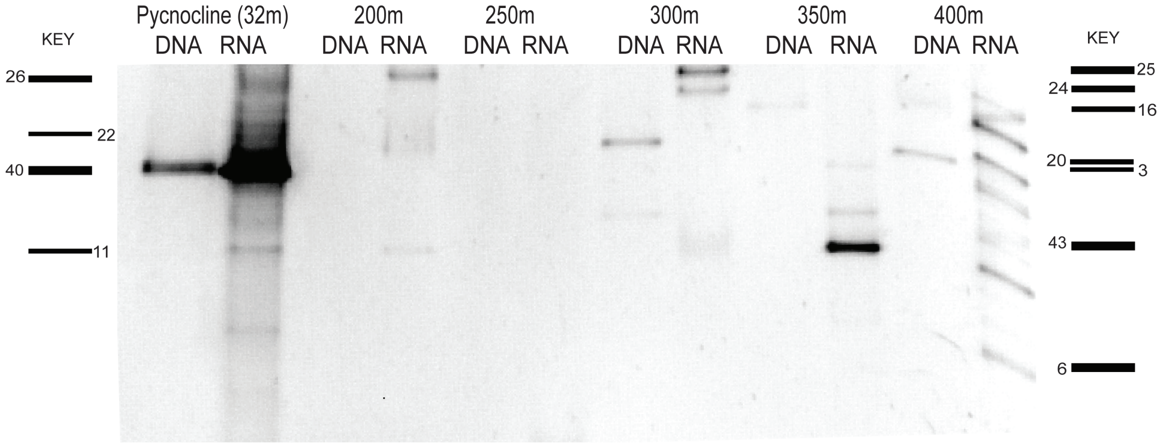

Figure S7. Offshore microsize (>10μm) DGGE of deep layers and pycnocline DNA and RNA samples. Key to the left shows DGGE Bands of lanes from the left-half through 300m DNA and the key to the right shows DGGE Bands of lanes from the right half, starting at 300m RNA through 400m RNA.

(A)

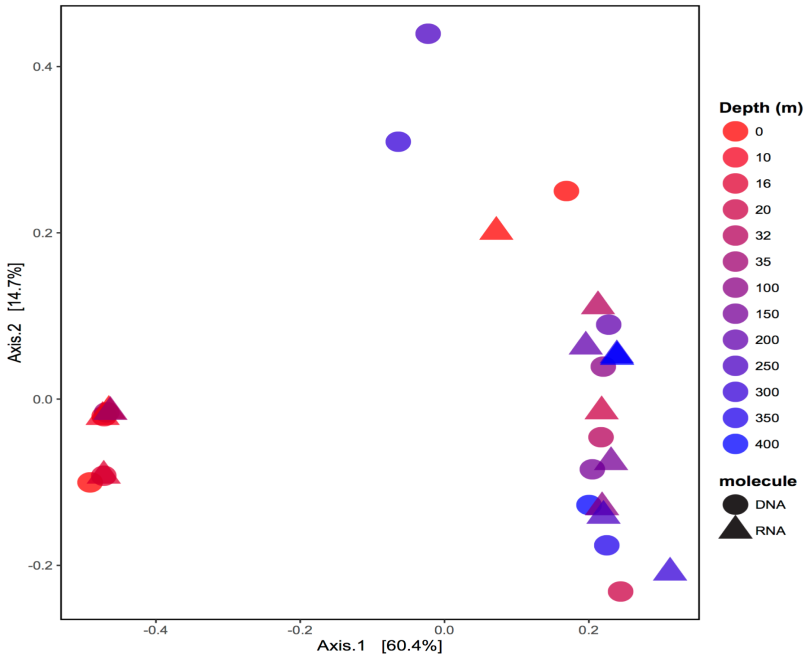

(B)

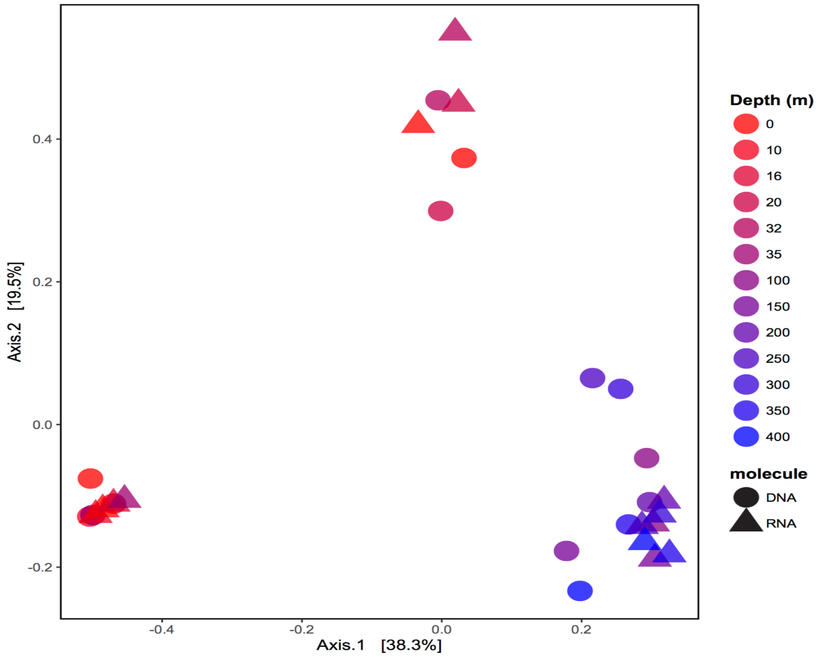

Figure S8. PCoA using **(A)** Unifrac dissimilarity and **(B)** Jaccard dissimilarity indices of samples from the nearshore and offshore stations with DNA and RNA analyzed separately. No clear pattern related to DNA or RNA is observed.
